# An Unusual Case of LGI1 (Leucine-Rich Glioma-Inactivated Protein 1) Limbic Encephalitis With Anti-acetylcholine Receptor and Anti-striational Autoantibodies

**DOI:** 10.7759/cureus.46491

**Published:** 2023-10-04

**Authors:** Akash Pathak, Jay Patel, Giselle Tran, Matthew Mrlik, Ning Zhong, Forshing Lui

**Affiliations:** 1 Neurology, California Northstate University College of Medicine, Elk Grove, USA; 2 Neurology, Kaiser Permanente Sacramento Medical Center, Sacramento, USA; 3 Clinical Sciences, California Northstate University College of Medicine, Elk Grove, USA

**Keywords:** adam22/23, caspr2, autoimmune antibodies, paraneoplastic syndrome, thymoma, lgi-1 limbic encephalitis, limbic encephalitis

## Abstract

Autoimmune encephalitis (AE) results from immune-mediated damage to the central nervous system (CNS) with varying clinical manifestations depending on autoimmune antibodies present and the antigens they target. Leucine-rich glioma-inactivated protein 1 (LGI1) has been recognized as one of the leading causes of limbic encephalitis (LE), presenting with seizures, memory loss, and faciobrachial dystonic seizures. A better understanding of the unique presentations of these AE allows for quick and effective diagnosis and treatment. We are presenting a very unusual case of LGI1 autoimmune LE with two additional autoantibodies, anti-acetylcholine receptor (AChR) and anti-striational, in a patient with an underlying thymoma. We will discuss the pathophysiology and common clinical presentation of anti-LGI1 autoimmune LE.

## Introduction

Autoimmune encephalitis (AE) are a diverse group of inflammatory central nervous system (CNS) conditions caused by immune-mediated damage to the brain. The clinical presentations of these conditions depend on the autoimmune antibodies present and the antigens they target [1-2]. 

Leucine-rich glioma-inactivated protein 1 (LGI1) is a highly expressed CNS protein with strongest expression in the hippocampus. The protein is secreted by neurons and functions transsynaptically to modulate both presynaptic and postsynaptic structures, including potassium channel Kv1.1 and glutamate receptor α-amino-3-hydroxy-5-methyl-4-isoxazolepropionic acid (AMPA). Dysregulation of these proteins by autoimmune damage to LGI1 results in the clinical manifestations of limbic encephalitis (LE) [1,3-4].

Anti-LGI1 antibody-mediated AE patients commonly present with seizures, memory loss, abnormal behavior, cognitive deficits, disorientation, dysautonomia, and hyponatremia [5]. The most commonly associated seizures include faciobrachial dystonic seizures, generalized tonic-clonic, and focal temporal lobe seizures. LGI1 LE is uncommonly associated with tumors. This case is unique due to the underlying thymic malignancy, the presence of anti-striational and anti-acetylcholine receptor (AChR) autoantibodies, and a lack of faciobrachial dystonic seizures and hyponatremia. This case adds to the small number of autoimmune LE case reports that present a patient with multiple autoimmune antibodies which indicates the presence of an underlying thymoma. The presence of AChR autoantibodies is highly specific for myasthenia gravis, which involves peripheral nervous system symptoms. However, no clinical evidence of myasthenia gravis was seen in our patient. This is quite interesting because anti-striational autoantibodies are often detected in a subset of myasthenia gravis patients with a thymoma. LGI1 LE is clinically rare and can be easily missed or misdiagnosed. Studying the different clinical manifestations of this disease in various cases allows for advancements in recognition and treatment, leading to a better prognosis.

## Case presentation

A 49-year-old female presented to the doctor’s office with palpitations with increasing frequency over the past few weeks and intermittent night terrors and dizziness for the last four days. One week prior, she experienced a brief loss of consciousness while driving, which led to a motor vehicle accident without any bodily injury. Since that episode, she was noted to have confusion, inability to recognize family members, and recent memory loss occurring in episodes lasting up to four hours and spontaneously resolving. The diagnosis of new-onset seizures was made and outpatient electroencephalography (EEG) was requested. Three weeks later, the patient presented to the emergency department for more episodes of loss of consciousness lasting 10 to 15 minutes with no warning signs, which were suspected as seizures by the emergency physician, and a neurology consult was requested. Clinical features of paranoia, language difficulty, and changes in behavior were also noted. The patient denied any shortness of breath or chest pain after experiencing palpitations for the previous few weeks.

Subsequent EEG showed intermittent polymorphic slow waves in the left temporal region and maximal waves in the anterior-mid temporal region lasting several seconds in duration with no electrographic seizures. Rarely, there was bilateral frontal and temporal slowing and less often intermittent right temporal polymorphic slowing. On one single occasion, there was an isolated left mid-temporal sharp wave. 

**Figure 1:**
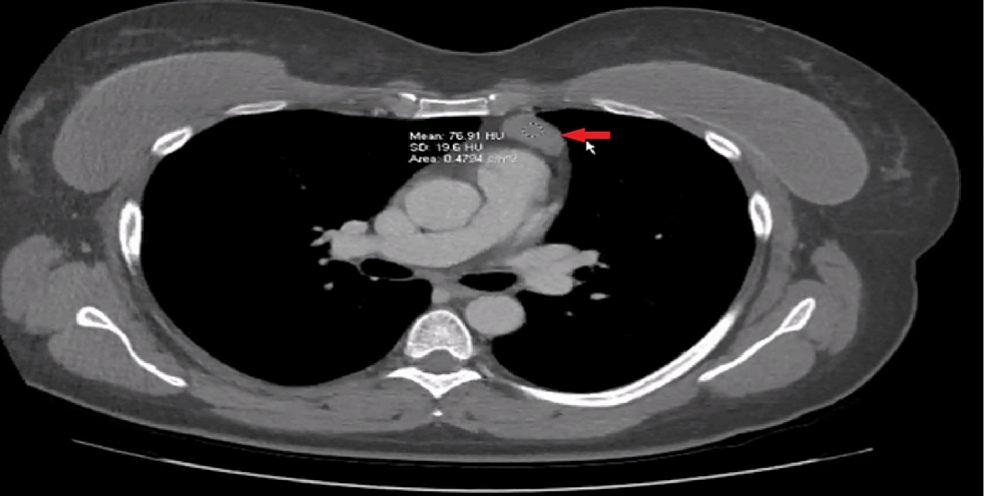
CT of chest showing thymoma (red arrow). CT, computerized tomography

A cerebrospinal examination was performed and showed borderline elevation of protein level. It revealed clear and colorless fluid with erythrocyte count (21 microL^-1^, reference range 0-5 microL^-1^), slightly elevated protein level (46 mg/dL, reference range 15-45 mg/dL), and presence of oligoclonal bands. Antibody testing was positive for anti-LGI1 receptor antibodies in cerebrospinal fluid and anti-striational and anti-AChR antibodies. Chest computerized tomography (CT) was performed and showed a thymoma (Figure 1).

**Figure 2:**
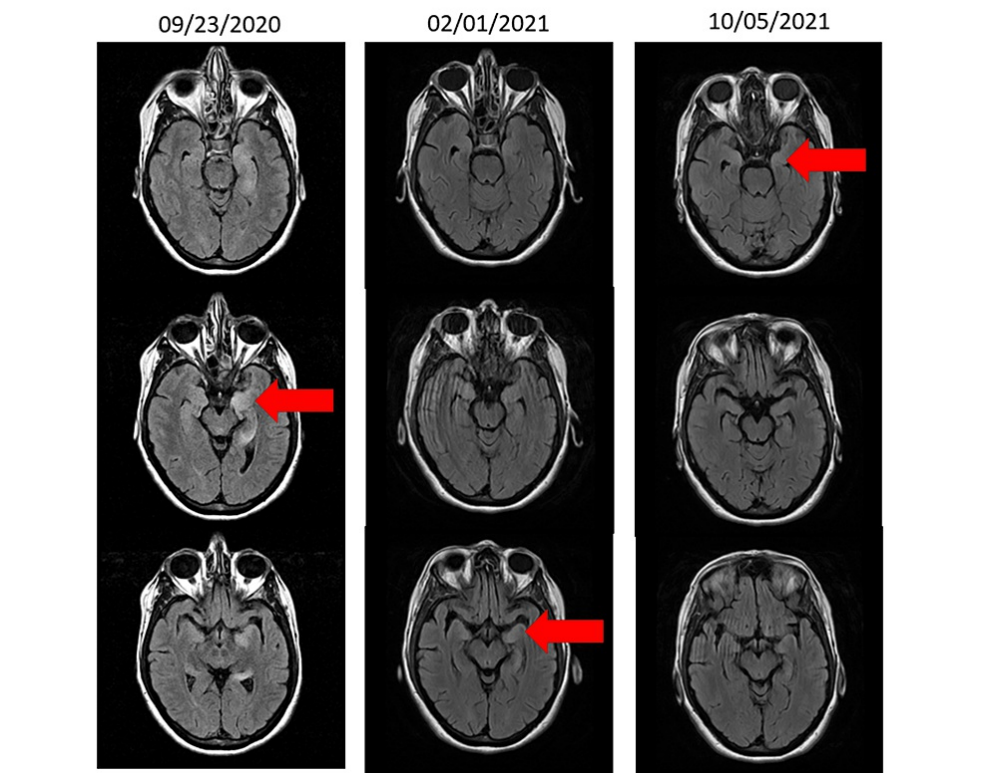
Serial brain magnetic resonance imaging (MRI) T2/FLAIR. 09/23/2020 shows hyperintensity in bilateral mesial temporal lobes, greater on the left side involving the hippocampus and fornix. 02/01/2021 shows subtle FLAIR hyperintensity in medial temporal lobes is mildly reduced compared with prior November imaging. 10/05/2021 shows mild residual T2 signal abnormality amygdala and hippocampi consistent with gliosis related to prior encephalitis. FLAIR, fluid-attenuated inversion recovery

Her brain fluid-attenuated inversion recovery (FLAIR) T2 signal MRI revealed bilateral mesial temporal lobe T2 hyperintensity greater on the left side involving the hippocampus and extending to the fornix (Figure 2). 

The patient was started on intravenous methylprednisolone (1 g/day) for five days followed by an oral prednisone taper in two weeks. The patient did not respond to the steroid regimen, and rituximab was started at an induction dose of 1 g by infusion over two doses two weeks apart. The patient again did not show any signs of improvement. She was then given five plasma exchanges followed by 2 g/kg intravenous immunoglobulin (IVIG) therapy over five days since the improvement was not satisfactory. Since then she has been placed on maintenance IVIG at the same dose every four weeks with slow and gradual improvement. All other treatments, including steroid, rituximab, and plasma exchanges, have been stopped since she was started on IVIG therapy. All her treatments were given in an outpatient facility, mostly in our infusion center. Her MRI performed after these treatments on February 21, 2021, showed subtle FLAIR hyperintensity in the medial temporal lobes. The finding was mildly improved compared to her previous MRI (Figure 2). The patient had a thymectomy performed. 

Follow-up one year later showed significant improvement from the previous status. However, pervasive declarative memory and neurocognitive profile deficits were noted. Visual recognition was an intact aspect of memory. Aspects of procedural memory were intact on testing and consistent with other aspects of procedural memory used by the patient in basic activities of daily living. Mild-to-moderate deficits in executive functioning were identified, specifically in identifying gestalt, planning and organizing, and retrieval of linguistic categories (5 percentile at best). Other skills, including inhibitory control, mental flexibility, problem-solving, and verbal reasoning, were only mildly below her baseline. Consistent with her residual clinical deficits, her brain MRI one year later (October 5, 2021) revealed mild residual T2 signal abnormality in the amygdala and hippocampi, consistent with gliosis related to prior encephalitis (Figure 2).

## Discussion

LGI1 LE most commonly presents with faciobrachial dystonic seizures, hyponatremia, memory loss, and cognitive deficits. One study of patients with LGI1 AE showed a 29% prevalence rate of faciobrachial dystonic seizures but was not seen in our patient [6]. Hyponatremia, which is present in 65% of cases, was also not seen in our patient. Rarely, the disorder has been associated with thymic tumors, and thymomas have been documented at a prevalence rate of less than 5% of LGI1 AE cases [7].

Paraneoplastic vs. non-paraneoplastic origin of AE

The many antibodies that cause AE can be classified based on their target antigens as well as their paraneoplastic or non-paraneoplastic origin. While underlying lung cancer is the most common cause of paraneoplastic AE, many other types of cancer can cause the disease, including testicular tumors, leukemia, lymphoma, and thymoma. Often, no clear etiology is found in patients with non-neoplastic AE, but some have been found to have an underlying autoimmune condition or to develop symptoms following a viral infection or vaccination [3]. The autoantibodies can be classified according to their target antigens. The two types of target antigens are cell surface/synaptic and intracellular antigens. The autoantibodies against cell surface/synaptic antigens are generally pathogenic through a B-cell-mediated immune mechanism, whereas the autoantibodies against intracellular antigens are not directly pathogenic and the immunopathogenic mechanism is more T-cell mediated [8]. The primary extracellular cell surface antigens include LGI1, contactin-associated protein-like 2 (CASPR2), gamma-aminobutyric acid-B (GABA-B) receptor, AMPA receptor, and N-methyl-D-aspartate (NMDA) receptor, and the intracellular antigens include Hu, Ma2, and others [9-10]. 

Of the many types of AE, autoimmune LE is the most common, affecting structures of the limbic system such as the hippocampus and medial temporal lobes. Since their discovery, antibodies against LGI1 have been recognized as the leading cause of LE, and the second most common cause of AE in general [2,11].

AChR and anti-striational antibodies

Our patient presentation is also unique because of the presence of two additional autoimmune antibodies, AChR and anti-striational, which typically indicate thymic malignancy. AChR antibodies target postsynaptic acetylcholine receptors found at the neuromuscular junction. Anti-striational antibodies target proteins such as titin and ryanodine receptors found in the cross-striations of skeletal and cardiac muscle [12]. The presence of AChR and anti-striational antibodies usually manifests as peripheral nervous system effects as commonly seen in myasthenia gravis, leading to symptoms such as weakness in the extremities and ptosis. AChR antibody is 99% specific for myasthenia gravis [13]. However, our patient experienced only CNS deficits suggesting that myasthenia gravis is not the likely etiology for the patient’s symptoms. This ties into the patient’s underlying thymoma as myasthenia gravis antibodies are produced in small amounts in the thymus [14]. The presence of multiple types of autoantibodies indicated a higher chance of an underlying malignancy [15].

Transsynaptic complex

A neuron’s intrinsic excitability at the neuromuscular junction is significantly influenced by Kv1 K+ channels, which line the presynaptic terminal. The Kv1 channels are associated with cell adhesion molecules (CAMs), which include LGI1 and CASPR2. CAMs alter the positioning of presynaptic and postsynaptic elements and are transported in vesicles along with the disintegrin and metalloproteinase domain-containing protein 22 (ADAM) proteins. By modulating the vesicular trafficking of CASPR2 and LGI1, ADAM22/23 leads to increased expression of the CAMs at the presynaptic neuron [16]. Thus, ADAM22 and ADAM23 proteins can influence the function of the transsynaptic complex and synaptic excitability. 

LGI1 is a secreted glycoprotein that links presynaptic potassium channels (Kv1.1) and postsynaptic AMPA receptors via the ADAM22/23 complex [17]. Dysregulation in this multi-protein scaffold induces abnormal neuronal excitability in hippocampal and temporal areas. LGI1 autoantibodies disrupt the interaction with ADAM proteins and thus affect the synaptic strength and excitability of Kv1.1 channels.

Another linkage protein that modulates the Kv channels is CASPR2, a CAM expressed highly in the limbic system and temporal lobe. Known to help cluster and position presynaptic Kv1 channels, the CASPR2 protein is a site of pathogenesis in AE. Anti-CASPR2 antibodies block the interaction with contactin-2, an important protein involved in forming neuronal connections, affecting the clustering and expression of Kv1 channels on the presynaptic membrane [18]. 

**Figure 3:**
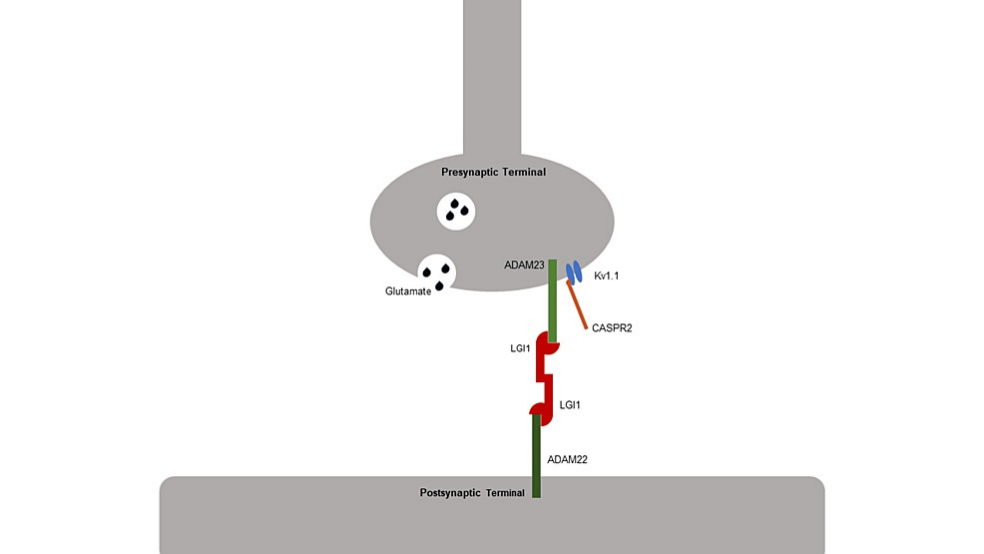
LGI1-ADAM22/23 transsynaptic complex. Image credits: Jay Patel. LGI1, leucine-rich glioma-inactivated protein 1; ADAM22, disintegrin and metalloproteinase domain-containing protein 22

The voltage-gated delayed rectifier potassium channel Kv1.1 plays a critical role in the regulation of neural excitability and is highly expressed in the hippocampus, cerebellum, and neocortex. Its blockade will result in a decreased action potential voltage threshold and increased action potential firing. The resulting dysfunction is linked to central and peripheral nervous system hyperexcitability and epilepsy. The channel’s activity is modulated by several mechanisms, including the LGI1, ADAM22/23, and CASPR2 proteins, which all together form a transsynaptic complex. A dysfunction in any of the components of the complex in the hippocampus has been implicated in epileptogenesis [19] (Figure 3).

Quick diagnosis of the disorder is necessary to curtail lasting memory deficits and cognitive deficits such as executive function. However, the numerous manifestations in patient presentations complicate diagnosis, which indicates the significance of early LGI1 antibody testing if LE is suspected. Continued follow-up is advisable as the two-year relapse rate is 27% [20].

## Conclusions

Anti-LGI1 LE is a prevalent cause of AE that has received substantial clinical attention over the past decade. While it is commonly characterized by faciobrachial dystonic seizures and hyponatremia, our case presented uniquely with the absence of both. In addition, our patient presented with two additional autoimmune antibodies, anti-AChR and anti-striational, which were concerning for an underlying malignancy and was later discovered to be a thymoma. Of note, our patient did not present with peripheral nervous system symptoms, despite anti-AChR’s high specificity for myasthenia gravis. This is quite interesting given the association between anti-striational and thymoma in subsets of patients with myasthenia gravis. Bringing light to this case allows for improved recognition of LGI1 LE, which is vital for early detection, correct diagnosis, and prompt initiation of treatment.
